# The Systemic Immune Inflammation Index as a Novel Predictive Biomarker for Contrast-Induced Acute Kidney Injury Risk Following Percutaneous Coronary Intervention: A Meta-Analysis of Cohort Studies

**DOI:** 10.2174/0115701611328810241028112700

**Published:** 2024-11-05

**Authors:** Yongqiang Zhang, Yong Xie, Chunyu Zhang, Jianglin Wang, Bin Liao, Jian Feng

**Affiliations:** 1 Department of Cardiology, The Affiliated Hospital of Southwest Medical University, Stem Cell Immunity and Regeneration Key Laboratory of Luzhou, Luzhou, Sichuan, China;; 2 Department of Cardiology, Hejiang County People's Hospital, Luzhou, Sichuan, China;; 3 Department of Pain Management, The Affiliated Hospital of Southwest Medical University, Luzhou, Sichuan, China;; 4 Department of Cardiovascular Surgery, The Affiliated Hospital of Southwest Medical University, Metabolic Vascular Diseases Key Laboratory of Sichuan Province, Luzhou, Sichuan, China

**Keywords:** Percutaneous coronary intervention, systemic immune inflammation index, contrast-induced acute kidney, injury, prognosis, meta-analysis

## Abstract

**Background:**

Contrast-induced Acute Kidney Injury (CI-AKI) frequently occurs as a complication following percutaneous coronary intervention (PCI), making the identification of high-risk patients challenging. While the systemic immune inflammation index (SII) might aid in predicting CI-AKI, the current evidence remains insufficient.

**Methods:**

We conducted a systematic literature search using PubMed, Web of Science, Embase, and the Cochrane Library, with a cut-off date of 3/20/2024. We included observational studies that examined the predictive value of SII for the risk of CI-AKI.

**Results:**

This meta-analysis encompassed 8 studies with a combined total of 6301 participants. Results showed pooled sensitivity and specificity of 0.73 (95% CI 0.69-0.76) and 0.68 (95% CI 0.57-0.77), respectively. The sROC curve analysis indicated an AUC of 0.74 (95% CI 0.70-0.78). The risk of publication bias was low (p = 0.18).

**Conclusion:**

The results of this study suggest that SII has a relatively high sensitivity and could function as a biomarker for the prediction of CI-AKI risk in people receiving PCI treatment.

## 1 INTRODUCTION

Studies have reported that the incidence of Contrast-induced Acute Kidney Injury (CI-AKI) in PCI patients ranges from 5% to 25% and is rising, now being the third leading cause of hospital-acquired acute kidney injury [1]. CI-AKI is generally a reversible condition and a significant complication of coronary angiography (CAG) and percutaneous coronary procedures [2, 3]. It is frequently linked to adverse clinical outcomes, such as prolonged hospitalization, progressive deterioration of renal function, and a relative increase in the risk of death [4-7]. Therefore, understanding the risk factors for secondary CI-AKI in percutaneous coronary intervention (PCI) patients is essential for developing effective prevention strategies and optimizing clinical management programs to improve patient outcomes and quality of life.

Studies have demonstrated that CI-AKI occurrence and progression are influenced by various mechanisms, such as inflammation, endothelial dysfunction, oxidative stress from reactive oxygen species, and renal vasoconstriction [8-10]. Despite an incomplete understanding of the underlying pathophysiological mechanisms of CI-AKI, activation of inflammation and immune responses has been found to play a central role [11-13]. Clinical observations and studies have validated that both the platelet-to-lymphocyte ratio (PLR) and the neutrophil-to-lymphocyte ratio (NLR) serve as reliable biomarkers for predicting the occurrence of contrast-induced acute kidney injury (CI-AKI) [14-16]. In 2014, Hu *et al*. proposed a more comprehensive inflammation assessment tool: the systemic immune inflammation index (SII), which is calculated as SII = (platelet × neutrophil) / lymphocyte [17]. This index evaluates immune and inflammatory status through a comprehensive analysis of neutrophils, platelets, and lymphocytes derived from routine complete blood count tests.

The SII has been demonstrated to be an independent prognostic indicator in various tumours [17-19]. In the cardiovascular system, SII also shows a significant positive correlation with patient mortality [20]. A study by Mert İlker Hayıroğlu *et al*. [21] found that higher SII is associated with long-term mortality and appropriate ICD therapy rates in heart failure patients receiving ICD treatment. Studies have also reported that SII is also a good predictor of the occurrence of CI-AKI after treatment with PCI [22]. Ali Bağcı found that the determination of SII before PCI treatment can help predict the risk of CI-AKI in ST-segment elevation my ocardial infarction (STEMI) patients [22]. Furthermore, Yang *et al*. further revealed that in patients undergoing PCI treatment, SII is more predictive of the occurrence of CI-AKI than well-known cardiovascular risk factors. However, despite promising prospects, research on SII and CI-AKI risk remains relatively limited [23]. Furthermore, there is no consistent conclusion regarding the accuracy of SII in the detection of CI-AKI after PCI. Therefore, in this diagnostic meta-analysis, we aimed to investigate whether SII could be used to predict the likelihood of developing CI-AKI in patients undergoing PCI and to provide guidance for subsequent clinical management.

## 2 METHODS

This study was conducted in accordance with the PRISMA guidelines [24] and registered in PROSPERO (CRD42024528495).

### 2.1 Search Strategy

The meta-analysis covered relevant articles on the diagnostic value of SII for PCI-induced CI-AKI. Articles were gathered from four English databases, namely Web of Science, PubMed, Embase, and the Cochrane Library, with a search cut-off date of March 20th, 2024, employing the subsequent keywords: (“SII” or “systemic immune-inflammation index”) and (“percutaneous coronary intervention,” “PCI” or “NSTEMI” or “STEMI” or “AMI”) and (“Contrast-Associated Acute Kidney Injury” or “CI-AKI” or “CA-AKI”). Searches were conducted in English databases without language restrictions. In addition to this, we further consulted the list of references for which relevant studies have been obtained in order to find more articles that meet the requirements for analysis.

### 2.2 Study Selection

Two investigators (ZYQ and XY) independently assessed the methodological quality of the included studies. In case of discrepancies, we documented and collaborated with a third investigator (ZCY) to address and resolve any disparities. To identify eligible articles, the following criteria were used: (1) original studies on patients undergoing PCI treatment; (2) studies examining the relationship between SII and PCI-induced CI-AKI; (3) provision of valid data related to CI-AKI diagnosis, such as sensitivity, specificity, positive predictive value, *etc*.; (4) article types include cohort studies or case-control studies; and (5) no language restriction.

(1) Reviews, preclinical studies, and studies not pertinent to the meta-analysis objective were excluded; (2) Animal experiments, conference proceedings, case reports, and publications with duplicate data were excluded; (3) Studies that did not demonstrate CI-AKI indicators after grouping by SII were also excluded.

### 2.3 Data Extraction and Quality Assessment

Firstly, duplicates were excluded, and the remaining search results underwent independent screening by two researchers. Titles and abstracts were meticulously reviewed using standardized inclusion and exclusion criteria. Finally, abstracts lacking sufficient information were selected for full-text analysis. Any disagreements were resolved by discussion or co-ordination.

Extracted data comprises: (1) The first author's name, country, and publication year; (2) The demographic characteristics of the patients, including the diagnosis, sample size, mean age, and the ratio of female to male patients; (3) Detailed information on AUC optimal cutoff value, specificity, sensitivity, and the related information with SII, along with the number of CI-AKI patients. The Quality Assessment of Diagnostic Accuracy Studies-2 (QUADAS-2) tool was used to determine the risk of bias, which is recommended for diagnostic study evaluation by the Cochrane Handbook [25]. The figures were plotted using RevMan 5.3 software. The green portion represents meeting the standard requirements, the red portion indicates not meeting the standard requirements, and the yellow portion indicates uncertainty.

### 2.4 Statistical Analysis

The following software was employed for data analysis in this research: Stata 15.1 and Meta-disc 1.4, with Meta-disc 1.4 software used to compute Spearman’s rank correlation coefficients of the logarithm of sensitivity and specificity to analyse the threshold effect. Meta-analysis was conducted using Stata 15.1, with heterogeneity analysis performed using Cochrane’s Q test, and heterogeneity was evaluated using the I2 index, where I2 scores of less than 25% were indicative of low heterogeneity, scores of 25% or higher but below 50% were suggestive of moderate heterogeneity, and scores of 50% or higher represented high heterogeneity [26]. Sensitivity and specificity indexes of each study were extracted. A comprehensive analysis was performed using a random effects model, which is considered a more general approach and is able to account for potential heterogeneity between included studies [27]. A comprehensive receiver operating characteristic (SROC) curve was plotted, and the area under the curve (AUC) was calculated; AUC> 0.70 or higher was regarded as a significant predictor of risk [28]. The Deeks' funnel plots were plotted to visualize publication bias. All statistical analyses were performed at a 0.05 significance level.

## 3 RESULTS

### 3.1 Study Selection and Study Characteristics

We examined four major databases and retrieved 594 relevant documents. Additionally, 2 articles were manually retrieved. Fig. (**1**) illustrates the study selection process flowchart and provides reasons for the exclusion of post-full-text reading. Firstly, reference management software (EndNote X7) was used to eliminate 324 duplicate publications. Then, 152 studies were removed because they were case reports, animal studies, commentaries, or reviews. Subsequently, 47 publications were selected to read and review in full. After further screening, 8 studies, encompassing 6301 participants, were considered for inclusion in the following meta-analysis [22, 29-35]. The median/mean age among the 6301 participants was between 54.68±10.10 and 68.79±9.48 years. The studies were carried out in two regions: China and Turkey. Regarding defining CI-AKI, 7 studies used an identical criterion, defining CI-AKI as a rise in the serum creatinine (Scr) level of 25% or 0.5 mg/dL above the baseline level within 48 to 72 hours. Another study delineated CI-AKI as an elevation in serum creatinine by a minimum of 50% or at least 0.3 mg/dL from baseline within 48–72 hours following contrast exposure. The AUC reflecting predictive model accuracy varied between 0.650 and 0.844. Sensitivity ranged from 66.8% to 77.6%, and specificity ranged from 53.1% to 84.5%. All studies reported threshold values for predicting CI-AKI occurrence, ranging from 586.19 to 1136. Detailed features of the included studies are mentioned in Table **1**.

### 3.2 Assessment of Quality

**Fig. (2):**
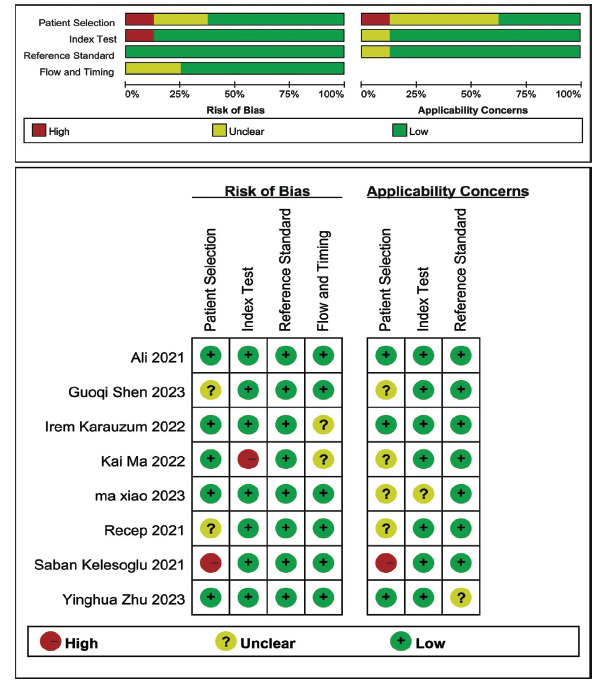
Quality assessment of included studies based on QUADAS-2 tool criteria.

Eight included articles were imported into RevMan 5.3 software one by one, and the QUADAS-2 scale was applied to assess the quality of the included literature. The results indicated that only patient selection was at high risk, while the other domains were at low risk, indicating that the 8 included articles in this study exhibited high quality (Fig. **2**).

### 3.3 Data Synthesis and Analysis

#### 3.3.1 Threshold Effect

The plane of the sROC curve did not show a “shoulder-arm” distribution. Further calculation of Spearman’s correlation coefficient (r = 0.071, P = 0.867) between sensitivity and log (1 - specificity) showed that there was no threshold effect, which was appropriate for combining sensitivity and specificity.

#### 3.3.2 The Diagnostic Accuracy of SII in Predicting CI-AKI

Concerning the diagnostic accuracy of SII, the combined sensitivity and specificity were 0.73 (95% CI: 0.69-0.76) and 0.68 (95% CI: 0.77-0.77), respectively. The I2 for sensitivity and specificity were 49.72% and 98%, as depicted in Fig. (**3**). Fig. (**4**) illustrates a combined positive likelihood ratio of 2.26 (95% CI: 1.67-3.06) and a combined negative likelihood ratio of 0.40 (95% CI: 0.33-0.48). The combined diagnostic odds ratio (DOR) shown in Fig. (**5**) was 5.62 (95% CI: 3.52-8.95). Lastly, Fig. (**6**) displays a combined AUC of 0.74 (95% CI: 0.70-0.78). These findings suggest that SII exhibits relatively high sensitivity but low specificity in diagnosing CI-AKI.

#### 3.3.3 Fagan Nomogram for Post-test Probabilities

**Fig. (7):**
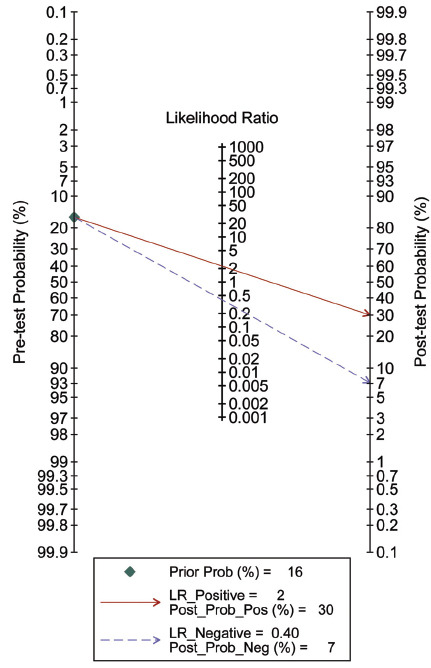
The clinical applicability of SII in forecasting the occurrence of contrast-induced nephropathy illustrated through Fagan’s nomogram plot.

The effectiveness of SII in predicting CI-AKI was assessed using a Fagan plot, with a PLR of 2 and an NLR of 0.40. Assuming an initial probability of CI-AKI occurrence of 16%, the positive test result using SII would adjust this probability to 30%, while the negative test result would adjust it to 7% (Fig. **7**).

#### 3.3.4 Sensitivity Analysis

**Fig. (8):**
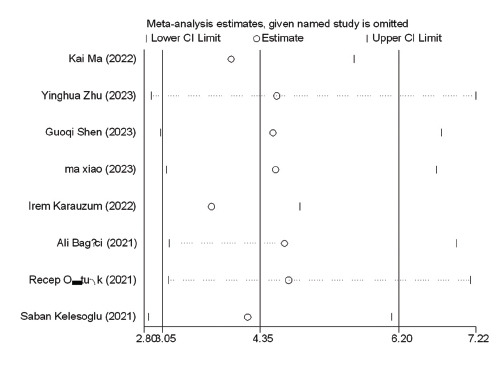
Sensitivity analysis.

The sensitivity analysis was carried out by excluding one study at a time. Our results demonstrated that no single study significantly affected DOR, further validating the integrity of the study (Fig. **8**).

#### 3.3.5 Publication Bias

**Fig. (9):**
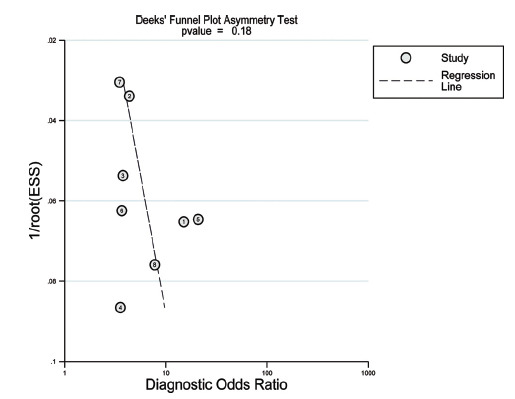
Funnel plot for publication bias assessment of included studies.

The Deek's funnel plot was used to test for asymmetry. No significant bias was found (P=0.18) (Fig. **9**).

## 4 DISCUSSION

This study was designed to assess SII diagnostic value in the prediction of CI-AKI in patients undergoing PCI. In terms of diagnostic accuracy, the SII's pooled sensitivity and specificity for predicting CI-AKI were 0.77 and 0.68, respectively, with an area under the SROC curve of 0.73. Based on the results of this study, the diagnostic accuracy of the SII for the prediction of CI-AKI was found to be moderate. Considering the significant heterogeneity among studies, caution is needed when interpreting and generalizing these findings.

As the world's population ages, the number of elderly patients in need of PCI treatment is on the rise. Older patients are more susceptible to CI-AKI due to reduced renal units, inadequate renal functional reserve, and multiple comorbidities. They are more likely to experience it and have a relatively worse prognosis [36, 37]. However, there is still uncertainty about the exact pathogenesis of CI-AKI. Research suggests that it may be related to renal vasoconstriction, inflammation, and direct cytotoxic effects of contrast agents [38, 39]. These factors may lead to increased inflammation in the body [40]. Animal and cell experiments have shown that injecting contrast medium (CM) can induce leukocyte infiltration into the kidneys, releasing pro-inflammatory cytokines and proteases, thereby exacerbating renal damage in experimental animals [41]. In addition, clinical studies have reported that some measures of inflammation, such as C-reactive protein (CRP), PLR, and NLR, have a strong association with the onset and development of CI-AKI [42]. SII, as a recently derived inflammatory biomarker, can simultaneously reflect aberrant activation of coagulation pathways and inflammatory pathways, both of which are potential mechanisms contributing to the onset and progression of CI-AKI. In comparison to other markers of inflammation, SII is a more sensitive predictor of inflammation in the body. Specifically, the overstimulation of inflammatory cells, such as neutrophils, further promotes the release of inflammatory mediators and the generation of reactive oxygen species, leading to decreased vascular permeability and endothelial dysfunction [43, 44]. Additionally, activated neutrophils elevate serum levels of arachidonic acid metabolites, thereby reducing the response to vascular dilation and causing vasoconstriction, all of which further lead to platelet adhesion and aggregation, resulting in renal capillary blockade. The result is a decrease in blood flow to renal tissue and exacerbation of ischemic injury [45]. Reduced lymphocyte levels can weaken the body's immune and antioxidant defenses, causing endothelial dysfunction and significantly contributing to renal tissue damage [46].

Growing research has highlighted the significant role of SII in diagnosing and prognosticating CI-AKI. Vladimir Shvartz *et al*. found that SII could predict the development of AKI in patients after aortic replacement operations [47], while Saban *et al*. found that the increase in SII in NSTEMI patients undergoing PCI was independently linked to the formation of CI-AKI [35]. A study by Jiang *et al*. found that SII may be the best inflammatory marker for predicting the risk of CI-AKI compared to other indices of inflammation [48]. Similarly, SII has been confirmed as a biomarker for AKI in some other clinical settings [49]. In 2021, Xu *et al*. found that the SII was able to predict the risk of acute kidney injury in patients with liver cancer after liver resection [50]. Lu *et al*. demonstrated that SII could precisely predict early-stage AKI in patients with severe pancreatitis [49]. These studies fully demonstrate the close relationship between SII and renal function.

Several risk scoring systems for the assessment of the likelihood of CI-AKI have been implemented in current years [51-53]. Notable examples among these are the Mehran score and the revised Mehran score 2 [54, 55]. However, the central role of inflammation in the development and progression of CI-AKI has not been recognised in previous CI-AKI risk score models, which did not include relevant inflammatory indicators [56]. Studies have reported that inflammation, circulating immune cells, and thrombosis play a significant part in the formation and maintenance of CI-AKI. Many inflammatory markers, including NLR and PLR, have been used to assess and predict CI-AKI [57, 58]. Kurtul *et al*. found that the development of CI-AKI in patients with NSTEMI undergoing CAG was positively correlated with the NLR [59]. Yalcin Velibey *et al*. [58] found that the elevated PLR levels correlated with a heightened risk of CI-AKI, with PLR capable of predicting CI-AKI occurrence in patients with STEMI undergoing initial PCI. Compared to PLR, SII reflects a broader inflammation status [23]. SII, being a composite of NLR and PLR, provides a more comprehensive evaluation of the correlation between CI-AKI and inflammation [60]. Additionally, artificial intelligence (AI) systems have demonstrated significant potential in accurately predicting AKI and other clinical endpoints [61, 62]. By processing and analyzing large amounts of complex data, AI can provide personalized risk assessments and precise treatment recommendations. Currently, the application of AI in coronary artery disease and atrial fibrillation has shown its important role in improving diagnostic and treatment decisions [63]. In the future, integrating AI technology with existing risk markers to further enhance the accuracy of CI-AKI prediction may be beneficial in optimizing patient treatment plans.

The present meta-analysis revealed that SII demonstrates a moderate diagnostic accuracy for the prediction of CI-AKI in patients receiving PCI, with comprehensive sensitivity and specificity values of 0.73 and 0.68, respectively, and the pooled AUC was 0.74. The diagnostic effectiveness of SII in predicting CI-AKI may stem from its incorporation of pivotal inflammatory markers, such as neutrophils, lymphocytes, and platelets, offering a comprehensive assessment of systemic inflammation. Given this mechanistic insight, future studies should strive to delineate the specific inflammatory pathways implicated in CI-AKI development and the interplay between SII and these pathways.

While the literature included in this study adjusted for potential confounding factors like hypertension, diabetes, and renal function, it did not consistently adjust for other factors, such as contrast agent volume and hemodynamic instability. The observed association between SII and CI-AKI may be reduced by missing mediating and moderating variables. Moreover, the variability in patient characteristics, SII thresholds, and CI-AKI definitions could diminish the predictive capacity of SII. Subsequent investigations ought to prioritize optimal adjustment for potential confounding factors to clarify the independent role of SII in CI-AKI onset.

There are some other limitations to this study: (1) we observed significant heterogeneity among the included studies despite the utilization of random-effects models, which may reduce the robustness of the conclusions. (2) The study subjects were limited to China and Turkey, so the applicability of these results to patients undergoing PCI treatment in other countries or regions may be limited. (3) The inclusion of only 8 original studies in this review is relatively limited. To increase the robustness of the evidence, further studies are needed to investigate the diagnostic and prognostic value of SII in CI-AKI.

## CONCLUSION

The findings of this meta-analysis revealed that SII exhibits relatively high sensitivity and holds promise as a biomarker for predicting CI-AKI risk in PCI patients. Nevertheless, the limited number of studies included in the analysis underscores the necessity for additional large-scale prospective studies in the future to further confirm the accuracy of SII predictions.

## Figures and Tables

**Fig. (1):**
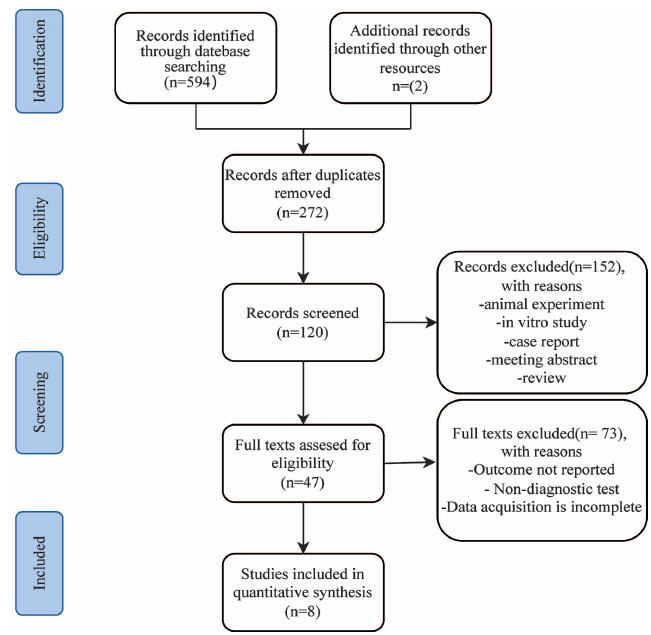
Flow diagram for study selection.

**Fig. (3):**
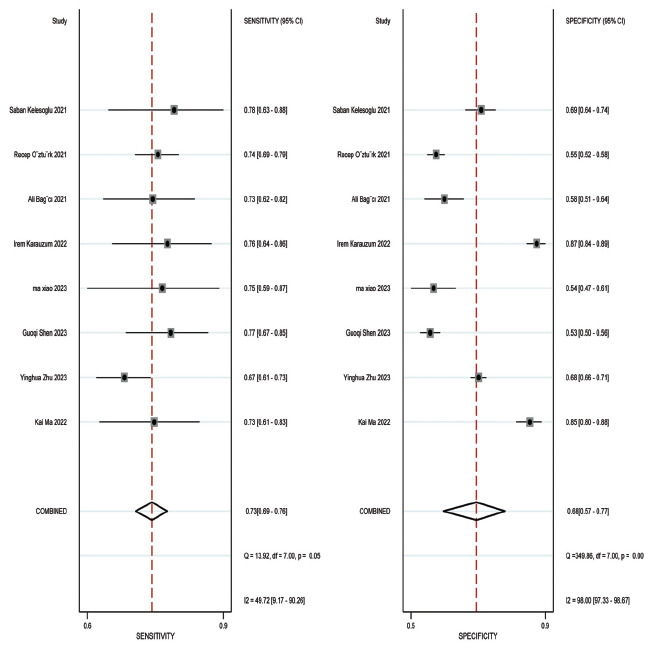
Forest plot depicting the combined sensitivity and specificity of the SII in predicting contrast-induced nephropathy.

**Fig. (4):**
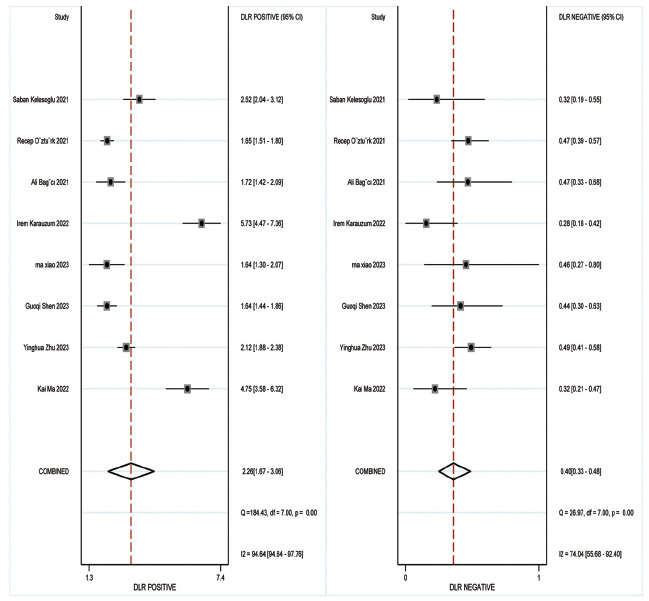
Forest plot depicting the likelihood ratio of the SII in predicting contrast-induced nephropathy.

**Fig. (5):**
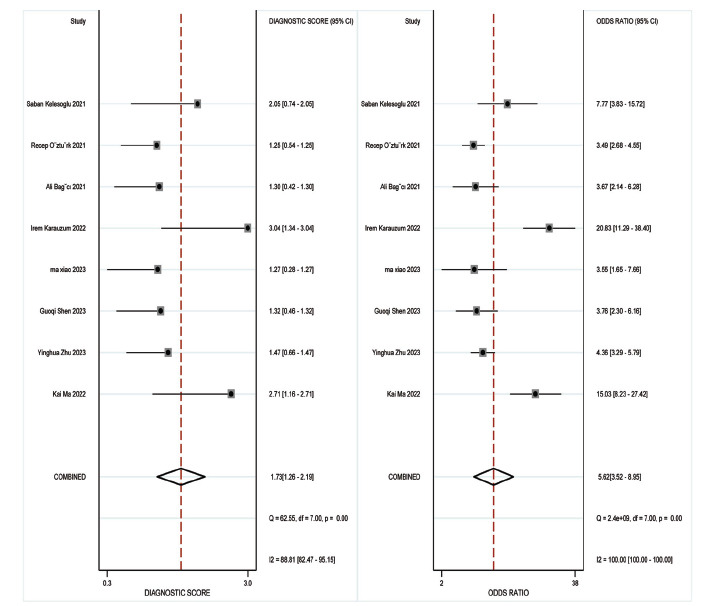
Forest plot depicting the diagnostic score and diagnostic odds ratio of the SII in predicting contrast-induced nephropathy.

**Fig. (6):**
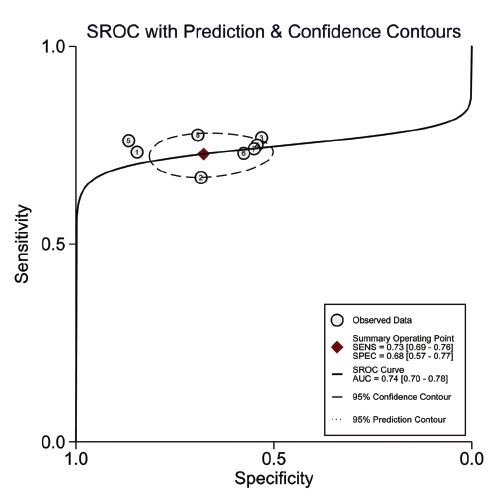
Analysis of summary receiver operating characteristic (sROC) curve demonstrating the predictive effectiveness of SII concerning contrast-induced nephropathy.

**Table 1:** Characteristics of studies (n = 8).

**Parameters**	**Kai Ma 2022 [** **29** **]**	**Yinghua Zhu 2023 [** **30** **]**	**Guoqi Shen 2023 [** **31** **]**	**ma xiao 2023 [** **32** **]**	**Irem Karauzum 2022 [** **33** **]**	**Ali Bag˘cı 2021 [** **22** **]**	**Recep O¨ztu¨rk 2021 [** **34** **]**	**Saban** **Kelesoglu 2021 [** **35** **]**
Country	China	China	China	China	Turkey	Turkey	Turkey	Turkey
Age (years)	62.70±12.92	68.79± 9.48	63.12±12.90	61.60±10.44	59.2±12.1	60.71±12.60	58.45±11.52	54.68±10.10
Male	324 (78.07%)	1084 (70.8%)	830 (76.50%)	193 (80.08%)	454 (71.8%)	283 (81.56%)	1312 (80.94)	326 (76%)
Hypertension	174 (41.93%)	788 (51.47%)	476 (43.87%)	164 (68.05%)	225 (35.6%)	145 (41.79%)	707 (43.62%)	205 (47.79%)
Diabetes mellitus	127 (30.6%)	377 (24.62)	287 (26.54%)	70 (29.05%)	200 (31.6%)	78 (22.48%)	422 (26.03%)	158 (36.83%)
Population	STEMI	ACS	STEMI	CAD	STEMI	STEMI	STEMI	NSTEMI
Sample	415	1531	1085	241	632	347	1621	429
CI-AKI	71 (17.11%)	259 (16.92%)	95 (8.76%)	40 (16.60%)	67 (10.6%)	85 (24.50%)	343 (21.16%)	49 (11.42%)
Non-CI-AKI	344	1272	990	201	565	262	1278	380
Definition of CI-AKI	An absolute serum creatinine increase ≥ 44 mol/L or a relative increase in serum creatinine ≥ 25% occurring within 48–72 h after the coronary procedure	An absolute serum creatinine increase ≥ 44 mol/L or a relative increase in serum creatinine ≥ 25% occurring within 48–72 h after the coronary procedure	Increase in serum creatinine of at least 50% or at least 0.3 mg/dL from baseline within 48–72 h after contrast exposure	An absolute serum creatinine increase ≥ 44 mol/L or a relative increase in serum creatinine ≥ 25% occurring within 48–72 h after the coronary procedure	An increase in the serum creatinine level of ≥0.5 mg/dL or ≥25% above baseline within 72 h after contrast medium exposure	A 25% increase or 0.5 mg/dL increase in absolute levels of creatinine 72 hours after the patient’s admission without any other etiology	Either a 25% increase in baseline serum creatinine levels or a 0.5 mg/dL increase in absolute serum creatinine levels within 72 hours of intravascular-CM administration without another etiology	As an increase in serum Cr by >0.5 mg/dL or > 25% within 72 hours after contrast medium administration
Cut-off values	831.05	736.08	1084.97	586.19	1282	735.56	1136	933.2
AUC	0.764	0.686	0.650	0.701	0.834	0.732	0.665	0.793
Sensitivity	73.2%	66.8%	76.8%	75%	76.1%	73.0%	74%	77.6%
Specificity	84.5%	66.3%	53.1%	54.2%	86.7%	57.5%	55%	69.2%

Abbreviations: ACS, Acute coronary syndrome; STEMI, STsegment elevation myocardial infarction; NSTEMI, Non-ST-segment elevation myocardial infarction; CAD,Coronary Artery Disease; CI-AKI, Contrast-induced acute kidney injury.

## Data Availability

All data generated or analyzed during this study are included in this published article.

## LIST OF ABBREVIATIONS

CAG: Coronary angiography
CI-AKI: Contrast-induced Acute kidney Injury
CM: Contrast medium
CRP: C-reactive protein
DOR: The combined diagnostic odds ratio
NLR: Neutrophil to lymphocyte ratio
PCI: Percutaneous coronary intervention
PLR: Platelet to lymphocyte ratio
QUADAS-2: The quality assessment of diagnostic accuracy studies-2
SII: Systemic immune inflammation index
SROC: Comprehensive receiver operating characteristic
STEMI: ST-segment elevation myocardial infarction

## STANDARD OF REPORTING

PRISMA guidelines were followed.
